# Investigating the Interrelationships Among Mental Health, Substance Use Disorders, and Suicidal Ideation Among Lesbian, Gay, and Bisexual Adults in the United States: Population-Based Statewide Survey Study

**DOI:** 10.2196/48776

**Published:** 2024-06-25

**Authors:** Alex Siu Wing Chan, Hon Lon Tam, Florence Kwai Ching Wong, Gordon Wong, Lok Man Leung, Jacqueline Mei Chi Ho, Patrick Ming Kuen Tang, Elsie Yan

**Affiliations:** 1 Department of Applied Social Sciences Faculty of Health and Social Sciences The Hong Kong Polytechnic University Hong Kong China; 2 Nethersole School of Nursing Faculty of Medicine The Chinese University of Hong Kong Hong Kong China; 3 Factor Inwentash Faculty of Social Work University of Toronto Toronto, ON Canada; 4 School of Clinical Medicine Department of Psychiatry The University of Hong Kong Hong Kong China; 5 School of Nursing Faculty of Health and Social Sciences Hong Kong Polytechnic University Hong Kong China; 6 Department of Anatomical and Cellular Pathology Prince of Wales Hospital, Faculty of Medicine The Chinese University of Hong Kong Hong Kong China

**Keywords:** mental health, adults, lesbian, gay, and bisexual, depression, drug abuse, drug dependence, suicidality risk, mental illness

## Abstract

**Background:**

Mental health disparities have been documented among lesbian, gay, and bisexual (LGB) adults in the United States. Substance use disorders and suicidal ideation have been identified as important health concerns for this population. However, the interrelationships among these factors are not well understood.

**Objective:**

This study aims to investigate the interrelationships among mental health, substance use disorders, and suicidal ideation among LGB adults in the United States using a population-based statewide survey.

**Methods:**

Our study was an observational cross-sectional analysis, and the data for this study were collected from a sample of LGB adults who participated in the statewide survey. The survey collected information on mental health, substance use disorders, and suicidal ideation using validated measures. Descriptive statistics and inferential data analysis were conducted to explore the interrelationships among these factors.

**Results:**

The results showed that LGB adults who reported higher levels of depression and drug abuse and dependence also reported higher levels of suicidal tendency and mental illness. Inferential data analysis using *χ*^2^ tests revealed significant differences in depression score (*χ*^2^_2_=458.241; *P*<.001), drug abuse and dependence score (*χ*^2^_2_=226.946; *P*<.001), suicidal tendency score (*χ*^2^_2_=67.795; *P*<.001), and mental illness score (*χ*^2^_2_=363.722; *P*<.001) among the 3 sexual identity groups. Inferential data analysis showed significant associations between sexual identity and mental health outcomes, with bisexual individuals reporting the highest levels of depression, drug abuse and dependence, suicidal tendency, and mental illness.

**Conclusions:**

This study provides important insights into the interrelationships among mental health, substance use disorders, and suicidal ideation among LGB adults in the United States. The findings underscore the need for targeted interventions and research aimed at addressing the mental health needs of sexual minority populations. Future research should aim to better understand the underlying mechanisms driving these disparities and develop culturally sensitive and tailored interventions that meet the unique needs of LGB individuals. Reducing stigma and discrimination against sexual minority populations is also crucial to improving their mental health outcomes.

## Introduction

Lesbian, gay, and bisexual (LGB) individuals encounter a multitude of health disparities stemming from their marginalized status and the strain of minority stress [1-3]. The focal points of concern encompass mental health, substance use disorders, and suicidal ideation within the LGB community [4-6]. Despite the recognition of these issues, there remains a discernible void in the comprehensive exploration of the intricate interplay among these concerns among LGB individuals within the United States.

Within the context of the LGB community, the nexus of mental health, substance use disorders, and suicidal ideation presents a complex landscape. Existing research underscores that LGB individuals are confronted with an elevated risk of grappling with mental health challenges—ranging from depression and anxiety to posttraumatic stress disorder—a consequence of societal stigma, discrimination, and violence [6-8]. The resultant minority stress often propels substance use disorders as a coping mechanism, manifesting in a higher prevalence of substance abuse and dependency among LGB individuals in contrast to the general population [9-11].

Furthermore, the vulnerability of LGB individuals to suicidal ideation and attempts surpasses that of the broader populace. The multifaceted risk factors underpinning suicidal ideation within this demographic encompass discrimination, victimization, familial or social rejection, internalized homophobia, and compromised mental health [12,13]. Remarkably, substance use disorders have been shown to further amplify the propensity for suicidal ideation and attempts among LGB individuals [5,14,15].

The intricate interlinking of mental health, substance use disorders, and suicidal ideation among LGB individuals is underscored by a nuanced interdependence [4]. For instance, compromised mental health may act as a precursor to substance use disorders, in turn fostering a milieu for the emergence of suicidal ideation. In a different trajectory, the direct influence of discrimination and victimization can accentuate both mental health adversities and heightened substance use [16-19]. In light of these multifaceted dynamics, a holistic approach to address mental health, substance use disorders, and suicidal ideation becomes imperative within the LGB community.

To this end, comprehensive interventions and policies targeting the underlying causative factors—discrimination and minority stress—are paramount to curtailing the prevalence of mental health issues, substance use disorders, and suicidal ideation among LGB individuals. Bridging this research gap, this study is dedicated to an in-depth exploration of the interrelationships among mental health, substance use disorders, and suicidal ideation among LGB adults within the United States. Leveraging a population-based statewide survey conducted in 2019 through an extensive web-based questionnaire, our study seeks to illuminate both the prevalence of these issues and the intricate associations binding them. Through these insights, we aspire to foster the development of efficacious interventions and policies designed to enhance the health and well-being of LGB individuals across the United States. The outcomes of this investigation are poised to advance the comprehension of the intricate interplay among mental health, substance use disorders, and suicidal ideation within the LGB community, thereby serving as a guiding compass for the implementation of evidence-based strategies to alleviate the burden of these issues within this population.

## Methods

### Setting

The study used data from the 2019 National Survey on Drug Use and Health (NSDUH), a nationally conducted population-based survey covering noninstitutionalized individuals across all 50 states within the United States.

### Population

The focus was on adults aged 35 years and above, resulting in a final sample size of 59,864 individuals.

### Sample

The sample consisted of individuals who participated in the 2019 NSDUH, a data set accessible through the web-based repository provided [20].

### Sampling

The NSDUH used a multistage probability sampling approach for each state to ensure a representative sample of the diverse population [21].

### Recruitment Process

Participants were selected through computer-assisted personal interviews and audio self-interviews, aiming to enhance the credibility of responses, especially on sensitive aspects of the survey. A remuneration was provided upon successful completion of the interview.

### Data Collection

The study used a comprehensive analysis of the NSDUH data set, covering domains such as mental health, substance use, and suicidal ideation.

### Data Analysis

The analysis involved the use of SPSS software (version 27.0; IBM). An observational cross-sectional analysis was conducted, using 4 derived variables: depression score, drug abuse and dependence score, suicidal tendency score, and mental illness score. These variables were calculated from specific questions within the survey, allowing a nuanced exploration of mental health within the LGB community [22]. Descriptive statistics in this study illustrated the distribution of depression scores, drug abuse and dependence scores, suicidal tendency scores, and mental illness scores across different sexual identities. The chi-square test of independence demonstrated the association between sexual identity and socioeconomic and health characteristics, revealing significant differences in total family income, poverty level, and overall health across sexual identity groups. Inferential data analysis indicated significant differences in mean ranks of depression score, drug abuse and dependence score, suicidal tendency score, and mental illness score across heterosexual, lesbian or gay, and bisexual groups.

### Variables

The meticulous assessment of mental health within the context of heterosexual, gay, and bisexual men in the United States engenders the use of 4 distinct derived variables [23].

#### Depression Score

Calculated through an intricate amalgamation of 8 questions sourced from the adult depression module of the survey. This module is broadly acknowledged in research circles for its efficacy in quantifying the severity of depressive symptoms and their reverberations upon an individual’s quality of life.

#### Drug Abuse and Dependence Score

This metric is derived through an intricate synthesis of 13 carefully curated questions, explicitly tailored to assess alcohol and drug abuse and dependence within the adult demographic. The strategic inclusion of this variable attests to the complex interrelationships between substance abuse and compromised mental health outcomes.

#### Suicidal Tendency Score

Forged from a set of 3 core questions, this variable delves into respondents’ perceptions and emotions surrounding suicide. Given the profound gravity of this issue within the realm of mental health, this variable emerges as a pivotal yardstick in evaluating the prevalence of suicidal tendencies within the study cohort.

#### Mental Illness Score

Rooted in the careful aggregation of 6 pivotal questions relating to the mental well-being of respondents over the preceding 12 months, this variable offers a comprehensive panorama of the overarching prevalence of mental illness within the study populace.

### Ethical Considerations

Ethical underpinnings stand resolutely as a cornerstone of our study. The international institutional review board at the esteemed Research Triangle Institute diligently oversees the National Drug and Alcohol Survey, a study purposefully sponsored by the Substance Abuse and Mental Health Services Administration. It is of notable significance that the institutional review board at the institution of the lead author has scrupulously evaluated and adjudged our analysis to be exempt from the purview of human participant research.

### Statement of Consent and Data Availability

This study procured its data from Opportunity Insights, a nonprofit organization situated in the United States. Opportunity Insights, located at Harvard University, specializes in using extensive data sets to convert scientific research into actionable policy changes.

## Results

### Descriptive Analysis

The descriptive analysis results (Table 1) show the distribution of depression score, drug abuse and dependence score, suicidal tendency score, and mental illness score across different sexual identities. The sample consisted of 59,864 individuals, of which 96.2% (n=57,575) identified as heterosexual, 1.7% (n=1017) identified as lesbian or gay, and 2.1% (n=1272) identified as bisexual.

**Table 1:** Descriptive statistics of depression, drug abuse and dependence, suicidal tendency, and mental illness scores by sexual identity.

Sexual identities	Respondents, n (%)	Depression score (range 0-1), mean (SD)	Drug abuse and dependence score (range 0-0.85), mean (SD)	Suicidal tendency score (range 0-2), mean (SD)	Mental illness score (range 0-5), mean (SD)
Heterosexual	57,573 (96.2)	0.5549 (0.22978)	0.0067 (0.03306)	0.4763 (0.24216)	0.5932 (1.14332)
Lesbian or gay	1038 (1.7)	0.6387 (0.25316)	0.0137 (0.05186)	0.5119 (0.30884)	0.9518 (1.44794)
Bisexual	1253 (2.1)	0.6972 (0.25646)	0.0208 (0.06673)	0.5890 (0.40123)	1.3113 (1.68115)
Total	59,864 (100)	0.5594 (0.23192)	0.0071 (0.03458)	0.4792 (0.24839)	0.6144 (1.16840)

The mean depression score for heterosexual individuals was 0.5549 (SD 0.22978), while the mean depression score for lesbian or gay individuals was 0.6387 (SD 0.25316), and for bisexual individuals it was 0.6972 (SD 0.25646). The mean drug abuse and dependence score for heterosexual individuals was 0.0067 (SD 0.03306), while the mean score for lesbian or gay individuals was 0.0137 (SD 0.05186), and for bisexual individuals it was 0.0208 (SD 0.06673). The mean suicidal tendency score for heterosexual individuals was 0.4763 (SD 0.24216), while the mean score for lesbian or gay individuals was 0.5119 (SD 0.30884), and for bisexual individuals it was 0.5890 (SD 0.40123). The mean mental illness score for heterosexual individuals was 0.5932 (SD 1.14332), while the mean score for lesbian or gay individuals was 0.9518 (SD 1.44794), and for bisexual individuals it was 1.3113 (SD 1.68115).

The results indicate that individuals who identified as lesbian, gay, or bisexual reported higher scores on all 4 variables compared to those who identified as heterosexual. The percentage of the total number for each sexual identity group was consistent across all 4 variables. The minimum and maximum scores for depression, drug abuse and dependence, and suicidal tendencies were 0 and 1, while the maximum score for mental illness was 5. The SD for all 4 variables was highest for bisexual individuals, indicating greater variability in scores for this group. The mean and SD of all 4 variables are presented for the overall sample, which can serve as a reference for future comparisons with other populations.

### Chi-Square Test of Independence

The chi-square test of independence was conducted to examine the association between sexual identity and 3 characteristics: total family income, poverty level, and overall health. The sample consisted of 59,864 individuals, of whom 96.2% (n=57,573) identified as heterosexual, 1.7% (n=1038) identified as lesbian or gay, and 2.1% (n=1253) identified as bisexual.

The results of the chi-square test of independence (Table 2) revealed that sexual identity was significantly associated with total family income (*χ*^2^_6_=97.704; *P*<.001), poverty level (*χ*^2^_4_=117.163; *P*<.001), and overall health (*χ*^2^_8_=32.945; *P*<.001). Specifically, higher proportions of LGB individuals reported lower total family income and higher poverty levels compared to heterosexual individuals. For example, 13.2% (n=7892) of individuals reporting a total family income of less than US $20,000 were heterosexual, while 0.3% (n=180) of individuals reporting the same income level were lesbian or gay and 0.4% (n=263) were bisexual. Similarly, 11.3% (n=6767) of individuals reporting living in poverty were heterosexual, while 0.2% (n=141) were lesbian or gay and 0.4% (n=244) were bisexual (Table 2). The associations between sexual identity and total family income and poverty level were statistically significant at the *P*<.001 level.

Regarding overall health in Table 2, there were statistically significant differences in the percentage of individuals reporting excellent, very good, good, fair, and poor health across sexual identity groups. However, the differences were relatively small, and the associations were statistically significant at the *P*<.05 level. Overall, these findings suggest that sexual identity is associated with socioeconomic status and health, with LGB individuals experiencing greater social and economic disadvantage compared to heterosexual individuals.

These additional results provide more detailed information about the distribution of each characteristic within and across sexual identity groups, which may be useful for understanding patterns of inequality and designing interventions aimed at reducing disparities.

**Table 2:** Association between sexual identity and socioeconomic and health characteristics (*χ*^2^ test results; N=59,864). Percentages represent comparisons within different groups and sexual identity categories in the data.

Characteristics				Heterosexual (n=57,573, 96.2%)		Lesbian or gay (n=1038, 1.7%)		Bisexual (n=1253, 2.1%)		Chi-square (*df*)	
**Total family income**											97.704 (6)^a^
	**Less than US $20,000 (n=8335)**										
		Total, n (%)	7892 (13.2)		180 (0.3)		263 (0.4)				
		Percentage within income group	94.7		2.2		3.2				
		Percentage within sexual identity	13.7		17.3		21				
	**US $20,000-US $49,999 (n=16,499)**										
		Total, n (%)	15,838 (26.5)		266 (0.4)		395 (0.7)				
		Percentage within income group	96		1.6		2.4				
		Percentage within sexual identity	27.5		25.6		31.5				
	**US $50,000-US $74,999 (n=9621)**										
		Total, n (%)	9265 (15.5)		165 (0.3)		191 (0.3)				
		Percentage within income group	96.3		1.7		2				
		Percentage within sexual identity	16.1		15.9		15.2				
	**US $75,000 or more (n=25,409)**										
		Total, n (%)	24,578 (41.1)		427 (0.7)		404 (0.7)				
		Percentage within income group	96.7		1.7		1.6				
		Percentage within sexual identity	42.7		41.1		32.2				
**Poverty level**											117.163 (4)^b^
	**Living in poverty (n=7152)**										
		Total, n (%)	6767 (11.3)		141 (0.2)		244 (0.4)				
		Percentage within income group	94.6		2		3.4				
		Percentage within sexual identity	11.8		13.6		19.5				
	**Income up to twice poverty line (n=11,067)**										
		Total, n (%)	10,625 (17.7)		150 (0.3)		292 (0.5)				
		Percentage within income group	96		1.4		2.6				
		Percentage within sexual identity	18.5		14.5		23.3				
	**Income more than twice poverty line (n=41,645)**										
		Total, n (%)	40,181 (67.1)		747 (1.2)		717 (1.2)				
		Percentage within income group	96.5		1.8		1.7				
		Percentage within sexual identity	69.8		72		57.2				
**Overall health**											32.945(8)^c^
	**Excellent (n=11,473)**										
		Total, n (%)	11,094 (18.5)		194 (0.3)		185 (0.1)				
		Percentage within health condition	96.7		1.7		1.6				
		Percentage within sexual identity	19.3		18.7		14.8				
	**Very good (n=21,091)**										
		Total, n (%)	20,297 (33.9)		370 (0.6)		424 (0.7)				
		Percentage within health condition	96.2		1.8		2				
		Percentage within sexual identity	35.3		35.6		33.9				
	**Good (n=18,235)**										
		Total, n (%)	17,523 (29.3)		314 (0.5)		398 (0.7)				
		Percentage within health condition	96.1		1.7		2.2				
		Percentage within sexual identity	30.4		30.3		31.8				
	**Fair (n=7311)**										
		Total, n (%)	6984 (11.7)		136 (0.2)		191 (0.3)				
		Percentage within health condition	95.5		1.9		2.6				
		Percentage within sexual identity	12.1		13.1		15.3				
	**Poor (n=1746)**										
		Total, n (%)	1670 (2.8)		24 (0)		52 (0.1)				
		Percentage within health condition	95.6		1.4		3				
		Percentage within sexual identity	2.9		2.3		4.2				

^a^Significant result: *P*<.001.

^b^Significant result: .001≤*P*<.01.

^c^Significant result: .01≤*P*<.05.

### Inferential Data Analysis

Inferential data analysis (Table 3) was conducted to determine whether there were significant differences in mean ranks of depression score, drug abuse and dependence score, suicidal tendency score, and mental illness score across 3 sexual identity groups: heterosexual, lesbian or gay, and bisexual. The sample consisted of 59,864 individuals, with 96.2% (n=57,599) identifying as heterosexual, 1.7% (n=1018) identifying as lesbian or gay, and 2.1% (n=1257) identifying as bisexual.

The results of the inferential data analysis revealed statistically significant differences in the mean ranks of all 4 variables across the 3 sexual identity groups. Specifically, heterosexual individuals had the lowest mean ranks for all 4 variables, while bisexual individuals had the highest mean ranks for all 4 variables. Lesbian or gay individuals had intermediate mean ranks for all 4 variables. The chi-square test of independence was used to determine the statistical significance of the differences in mean ranks. The results of the chi-square tests were statistically significant for all 4 variables (depression score: *χ*^2^2=458.241; *P*<.001; drug abuse and dependence score: *χ*^2^2=226.946; *P*<.001; suicidal tendency score: *χ*^2^2=67.795; *P*<.001; and mental illness score: *χ*^2^2=363.722; *P*<.001). The results suggest that there are significant differences in the distribution of depression score, drug abuse and dependence score, suicidal tendency score, and mental illness score across sexual identity groups.

These findings suggest that sexual identity is an important factor to consider when examining depression, drug abuse and dependence, suicidal tendency, and mental illness. The findings also underscore the importance of addressing the specific mental health needs of sexual minority populations.

**Table 3:** Mean ranks of depression score, drug abuse and dependence score, suicidal tendency score, and mental illness score by sexual identity.

Characteristics			Mean ranks						
			Depression score		Drug abuse and dependence score		Suicidal tendency score		Mental illness score
**Sexual identity**									
	Heterosexual (straight)	29,650.64		29,845.84		29,846.33		29,731.5	
	Lesbian or gay	35,164.76		31,287.05		30,820.18		33,393.78	
	Bisexual	38,549.02		32,792.46		33,156.47		36,300.86	
Chi-square (*df*)			458.241 (2)^a^		226.946 (2)^b^		67.795 (2)^c^		363.722 (2)^c^

^a^Significant result: *P*<.01.

^b^Significant result: .001≤*P*<.01.

^c^Significant result: .01≤*P*<.05.

## Discussion

### Principal Results

In recent years, there has been growing recognition of the unique mental health needs of LGB individuals. Studies have consistently shown that sexual minority populations are at increased risk for mental health problems including depression, anxiety, substance use disorders, and suicidal ideation. These disparities have been attributed to a range of factors, including discrimination, stigma, and marginalization, as well as social and economic disadvantage [24-26].

The results of this study support these findings, demonstrating that sexual minority individuals in the United States are at greater risk for experiencing social and economic disadvantage, as well as mental health problems, compared with heterosexual individuals [27]. The descriptive statistics analysis revealed that LGB individuals were more likely to report lower total family income and higher poverty levels compared with heterosexual individuals. These differences were statistically significant, indicating a robust association between sexual identity and socioeconomic status. Furthermore, the chi-square test of independence demonstrated that a higher percentage of LGB individuals reported lower total family income and higher poverty levels compared with heterosexual individuals. These findings suggest that sexual minority populations experience greater social and economic disadvantage compared with heterosexual individuals, which may contribute to their increased risk for mental health problems.

Moreover, the inferential data analysis demonstrated that sexual minority individuals are at increased risk for mental health problems compared with heterosexual individuals. Bisexual individuals, in particular, had the highest mean ranks for depression score, drug abuse and dependence score, suicidal tendency score, and mental illness score. These findings underscore the need for targeted interventions to address the mental health needs of sexual minority populations, particularly those who identify as bisexual.

The mental health disparities experienced by sexual minority populations have important implications for health care providers, policy makers, and other stakeholders. These disparities must be recognized and addressed through the development of targeted interventions aimed at reducing stigma and discrimination, increasing access to mental health services, and addressing the social and economic factors that contribute to poor mental health outcomes among sexual minority individuals [13,28-30]. Health care providers can play a critical role in addressing the mental health needs of sexual minority populations by creating safe and supportive environments that are free from discrimination and stigma [31-34]. This may involve providing culturally sensitive care, offering support groups or counseling services that are specifically tailored to the needs of sexual minority individuals, and increasing access to mental health services through community-based organizations and other resources.

Policy makers can also play an important role in addressing the mental health needs of sexual minority populations. This may involve passing laws and policies that protect the rights of sexual minority individuals such as antidiscrimination laws and laws that ensure access to health care and other resources [35,36]. Additionally, policy makers can work to increase funding for research and intervention programs aimed at reducing mental health disparities among sexual minority populations [37]. The results of this study highlight the importance of addressing the unique mental health needs of sexual minority populations. The findings suggest that sexual minority individuals are at increased risk for experiencing social and economic disadvantage, as well as mental health problems, compared with heterosexual individuals [38]. Health care providers, policy makers, and other stakeholders must work together to develop targeted interventions aimed at reducing these disparities and ensuring that all individuals have access to the support and care they need to lead healthy and fulfilling lives.

### Future Implications

The findings of this study have important implications for future research and intervention efforts aimed at addressing the mental health needs of sexual minority populations. The following section outlines several potential future directions for research and intervention in this area.

First, future research should aim to better understand the underlying mechanisms driving the mental health disparities experienced by sexual minority populations. This may involve investigating the role of discrimination, stigma, and marginalization in contributing to poor mental health outcomes among sexual minority individuals. Additionally, future research could explore the impact of other social and environmental factors, such as social support and access to health care, on mental health outcomes among sexual minority populations.

Second, future intervention efforts should aim to address the specific mental health needs of sexual minority populations. This may involve developing culturally sensitive and tailored interventions that are designed to meet the unique needs of LGB individuals. For example, interventions could include support groups or counseling services that are specifically tailored to the needs of sexual minority individuals, as well as community-based programs that provide access to mental health services and other resources.

Third, future interventions should also focus on reducing stigma and discrimination against sexual minority populations. This may involve working with health care providers and policy makers to develop and implement policies and programs that protect the rights of sexual minority individuals, increase access to health care and other resources, and reduce discrimination and stigma in health care settings.

Finally, future research and intervention efforts should also focus on the mental health needs of bisexual individuals. The results of this study suggest that bisexual individuals may be at particularly high risk for poor mental health outcomes, compared with other sexual minority populations. Therefore, interventions aimed at addressing mental health disparities among sexual minority populations should include a specific focus on the needs of bisexual individuals.

The findings of this study underscore the need for targeted interventions and research aimed at addressing the mental health needs of sexual minority populations. By developing culturally sensitive and tailored interventions, reducing stigma and discrimination, and addressing the specific needs of bisexual individuals, we can work to reduce mental health disparities and ensure that all individuals have access to the support and care they need to lead healthy and fulfilling lives.

### Limitations

The primary focus of this study revolves around examining the intricate relationships between various variables within the context of mental health, substance use disorders, and suicidal ideation in LGB adults. However, it is important to acknowledge that our investigation does not extend to the assessment of specific interventions targeting these issues. Consequently, although we have successfully discerned associations between the examined variables, our study is unable to furnish insights into the effectiveness of interventions aimed at ameliorating mental health concerns, substance use disorders, or suicidal ideation among LGB adults.

This limitation not only emphasizes the scope of our study but also underscores the necessity for future research endeavors to venture into the domain of interventions. To comprehensively address the challenges faced by the LGB community in terms of mental health and well-being, further investigations should explore and evaluate the potential impact and efficacy of intervention strategies. By delving into this unexplored territory, future studies can contribute to a more comprehensive understanding of effective approaches for reducing mental health disparities, substance use issues, and suicidal tendencies within the LGB population.

### Conclusion

This study highlights the need for future research and interventions that address the mental health needs of sexual minority populations. Future research should investigate the underlying mechanisms driving mental health disparities and explore the impact of social and environmental factors on mental health outcomes. Interventions should be developed that address the specific needs of sexual minority populations including tailored support groups and community-based programs. Efforts to reduce stigma and discrimination against sexual minority individuals are also crucial. Finally, interventions should focus on the mental health needs of bisexual individuals, who may be at particularly high risk for poor mental health outcomes. By addressing these issues, we can work toward reducing mental health disparities and ensuring that all individuals have access to the support and care they need.

## Abbreviations

LGB: lesbian, gay, and bisexual
NSDUH: National Survey on Drug Use and Health

## Data Availability

The data sets generated or analyzed during this study are available in the 2019 National Survey on Drug Use and Health (NSDUH) repository [20].
